# Rumen microbiota associated with feed efficiency in beef cattle are highly influenced by diet composition

**DOI:** 10.1016/j.aninu.2024.11.027

**Published:** 2025-03-15

**Authors:** Abimael Ortiz-Chura, Karla Fabiola Corral-Jara, Jeremy Tournayre, Gonzalo Cantalapiedra-Hijar, Milka Popova, Diego P. Morgavi

**Affiliations:** Université Clermont Auvergne, INRAE, VetAgro Sup, UMR 1213 Herbivores Unit, Saint-Gènes-Champanelle, France

**Keywords:** Residual feed intake, Rumen microbiome, Metatranscriptomics, Metataxonomics

## Abstract

Given the role of the rumen microbiome in providing nutrients to the host ruminant, it is expected that rumen microbes contribute to inter-animal variations in feed efficiency. However, the link between microbial structure and an “efficient” host phenotype is unclear. We hypothesized that extreme residual feed intake (RFI) phenotypes would display distinctive microbiome features regardless of the diet. In this study, we selected the 32 most extreme RFI Charolais bulls from a cohort of 100 animals fed corn-silage (CS; *n* = 50) or grass-silage (GS; *n* = 50) based diets. Rumen samples were obtained 3 h after feeding, at slaughter, for fermentation and metataxonomic and metatranscriptomic microbial analysis. Volatile fatty acid profiles showed no differences between diets and between extreme RFI phenotypes (*P* > 0.05). Total bacteria and methanogen populations did not differ between extreme RFI phenotypes (*P* > 0.05), although methanogens expressed per liquid rumen digesta weight tended to decrease in the most efficient bulls compared to the least efficient ones (*P* = 0.10). The rumen microbial community structure differed between diets (*P* < 0.001), and between extreme RFI phenotypes in the GS diet. In the whole dataset, we identified *Succiniclasticum*, *Saccharofermentans*, *Clostridia_258483* and CAG-238 as bacteria discriminant between extreme RFI phenotypes (*q* < 0.10). Within diets, these four genera were also discriminant in the GS diet and were all associated with the least efficient bulls. In contrast, in bulls fed the CS diet, only *Saccharofermentans* and *Succiniclasticum* were discriminant but they were positively associated with the most efficient bulls. Rumen microbial functional features did not differ between extreme RFI phenotypes but did differ between diets. In conclusion, the rumen microbiome was mainly influenced by diet, with the RFI phenotype being a marginal effector. *Succiniclasticum*, *Saccharofermentans*, *Clostridia_258483*, and CAG-238 were discriminant between extreme RFI phenotypes regardless of diet. However, the direction of the association with RFI was diet dependent, indicating a diet-RFI interaction and suggesting that these discriminating microbes may be suitable microbial indicator species for RFI only when considered in conjunction with the diet information.

## 1 Introduction

Ruminant production is pivotal for global food security, and global beef consumption is projected to increase from 70 to 76 million tonnes in 2031 compared to 2021. This expected higher demand is primarily driven by the projected population growth to 8.6 billion by 2031 (OECD/FAO, 2022). The challenge for the beef cattle industry is to meet this growing demand without increasing the global cattle herd. To achieve this, it is necessary to improve the productive, economic and environmental efficiency of ruminant production systems.

Given that feeding cost represents approximately 70% of variable costs in livestock production (Makkar, 2018), there is a growing interest in improving feed efficiency. Residual feed intake (RFI) is a measure of feed efficiency that has the potential to reduce production costs as the variation in feed intake between similarly performing animals but with extreme RFI phenotypes (low RFI = most efficient and high RFI = least efficient) differs from 15% to 34% in beef cattle (Liu et al., 2022; Lourenco et al., 2022; Welch et al., 2021). Similarly, several studies have linked RFI with environmental sustainability. In particular, a positive correlation has been observed between RFI and methane (CH_4_) production (g/d) (Alemu et al., 2017; Bes et al., 2022; Manzanilla-Pech et al., 2022). However, this correlation is not consistently observed across all studies, as some have reported no correlation between RFI and CH_4_ (g/d), or even a negative correlation with CH_4_ yield (g/kg DMI) in beef cattle (McDonnell et al., 2016; Renand et al., 2019).

The rumen microbiome plays a fundamental role in feed utilization and nutrient provision to the host ruminant. It is therefore expected that the rumen microbiome may contribute to inter-animal variations in feed efficiency. However, establishing consistent patterns of association between microbial structure and an “efficient” host phenotype has proven challenging (Lopes et al., 2021; McGovern et al., 2020). Possible explanations for the heterogeneity or lack of responses could be the type of diet, which is known to be the primary factor influencing the microbial community (Huws et al., 2018) as well as the influence of time of sampling after feeding and intake (Cantalapiedra-Hijar et al., 2018) that is not uniform among studies. The host genotype also plays a role (Hernandez-Sanabria et al., 2013); and variables related to sample collection, sample processing, and data analysis methods may influence results (Nearing et al., 2022).

It is worth noting that most of the aforementioned studies have focused on taxonomic profiles, and the relationships between the metabolic functions of the rumen microbiota and their role in feed efficiency remain unclear. Studies using a metatranscriptomics approach effectively correlated rumen microbial profiles with feed efficiency in beef cattle fed high-energy fattening diets (Li et al., 2019; Li and Guan, 2017). These studies reported less diverse functional microbiome in the rumen of the most efficient bulls compared to the least efficient ones. However, these studies did not investigate whether the same response would occur with different diets. Moreover, it has been observed that shifting from a forage-rich to a concentrate-rich diet can lead to changes in how animals are classified in terms of RFI (Lahart et al., 2020). This raises the question of whether the rumen microbiome could underlie this interaction between diet and RFI classification. Further exploration in this area is necessary to gain a better understanding of these complex relationships.

We hypothesized that extreme RFI phenotypes in beef cattle would display distinctive taxonomic and functional features of the rumen microbiome regardless of diet. To investigate this, we utilized metataxonomics and metatranscriptomics approaches to examine the relationship between the extreme RFI phenotypes and the taxonomic and functional profiles of the rumen microbiome in fattening Charolais bulls fed two contrasting diets.

## 2 Materials and methods

### 2.1 Animal ethic statement

This experiment was conducted at INRAE, Centre Auvergne-Rhône-Alpes, France. The protocol of this study was approved by the Ethics Committee of the Auvergne-Rhône-Alpes region and the French Ministry of Higher Education, Research and Innovation (authorization number: APAFIS #16194-2016101016361277 v6 delivered on 14th January 2019).

### 2.2 Experimental design and diet composition

This experiment was carried out as a part of a large study designed to evaluate biological determinants of feed efficiency in extreme RFI young bulls fed two contrasting diets (Bes et al., 2022; Guarnido-Lopez et al., 2023, 2022). Briefly, the study consisted of a cohort of 100 growing Charolais bulls (382 ± 41 kg live weight and 259 ± 42 days old at the start of the experiment) that were fed corn silage (CS)- or grass silage (GS)-based diets from two independent batches (experimental years 2019 [*n* = 50 bulls] and 2020 [*n* = 50 bulls]). Following a four-week period of adaptation to the facilities and their experimental diet, the feed efficiency of all bulls was evaluated over an 84-day period. In each batch (experimental year), the results of this feed efficiency test allowed for the classification of the bulls within each diet according to their RFI value, resulting in a total of 32 most extreme bulls (four for each batch and diet).

For each batch, the RFI was calculated as a difference between the dry matter intake (DMI) observed and expected, i.e., the residual of the regression equation of DMI observed with average daily gain (ADG) and average metabolic body weight (metabolic BW = BW^0.75^) adjusted for the effect of diet, as follows:

$$\text{Observed}\;\text{DMI}=\beta_{0}+D+\beta_{1}\;\left(\text{metabolic}\;\text{BW}\right)+\beta_{2}\;\left(\text{ADG}\right)+e\text{,}$$

where *β*_*0*_ is the intercept, *D* is the effect of diet, *β*_*1*_ is the regression coefficient for metabolic BW, *β*_*2*_ is the regression coefficient for ADG, and *e* is the residual of the model or RFI.

Experimental diets were based either on CS or GS and concentrate (Table 1). Diets were distributed daily in the morning as total-mixed rations and the animals were fed individually and ad libitum. All feed samples from the entire fattening period were analyzed for DM, organic matter (OM), total nitrogen (TN), neutral detergent fiber (NDF), acid detergent fiber (ADF) and starch, as described in Guarnido-Lopez et al. (2022). Briefly, DM and OM concentrations were determined by oven-drying at 60 °C for 72 h, and subsequent incineration at 550 °C for 4 h (method 942.05; AOAC, 2005). Total nitrogen concentration was determined using the Dumas method (method 968.06; AOAC, 2005) and the crude protein (CP) was calculated as TN × 6.25. The concentrations of NDF and ADF were determined as described by Van Soest et al. (1991). The starch concentration was analyzed using the enzymatic method (Faisant et al., 1995). The NDF and ADF concentrations of all feed ingredients were analyzed; whereas only the enzymatic degradability of CP and the starch concentration of concentrate ingredients were analyzed. Feed values for individual ingredients and experimental diets were calculated according to (INRA, 2018).

**Table 1:** Dietary ingredients, chemical composition and feed values of the experimental diets offered to fattening Charolais young bulls.

Item	GS	CS
**Ingredient composition, % of DM**		
Corn silage		62.1
Grass silage	59.20	
Wheat straw	5.08	4.08
Wheat grain	7.68	20.8
Beet pulp	23.7	
Soybean meal	3.93	11.5
Bicarbonate		0.67
Mineral and vitamin mix^1^	0.38	0.3
Total	100.00	100.00
**Chemical composition**,^2^**g/kg of DM**		
Organic matter	883 ± 5.75	955 ± 1.56
Crude protein	141 ± 4.94	140 ± 8.48
Neutral detergent fiber	454 ± 37.5	331 ± 1.41
Acid detergent fiber	254 ± 21.2	158 ± 10.6
Starch	48.5 ± 4.90	329 ± 1.41
Starch/NDF, g/g	0.11 ± 0.02	0.99 ± 0.01
**Calculated values**^3^		
Net energy, MJ/kg of DM	6.3	6.95
Rumen CP degradability^4^, %	74.8	72.8
MP, g/kg of DM	82.0	84.9
RPB^5^, g/kg of DM	9.00	4.00

GS = grass silage; CS = corn silage; DM = dry matter; NDF = neutral detergent fiber; CP = crude protein; MP = metabolizable protein; RPB = rumen protein balance.

1 Minerals and vitamin mix: 5% P, 25% Ca, 8% Mg, 0.2% Na, vitamin A (210 mg/kg), vitamin D_3_ (3.75 mg/kg), vitamin E (2730 mg/kg) and vitamin B_1_ (4.5 mg/kg).

2 Means ± standard deviations of chemical composition (*n* = 2) of experimental diets from two consecutive years (2019 and 2020).

3 Feed values were estimated from chemical composition and proportion of ingredients considering digestive interactions (INRA, 2018).

4 Estimated from an enzymatic hydrolysis assay for the concentrate (Aufrère and Cartailler, 1988) or from chemical composition for forages (INRA, 2018).

5 The RPB, representing nitrogen intake minus non-ammonia nitrogen at the duodenum (INRA, 2018).

### 2.3 Rumen sampling and fermentation profiling

Rumen samples were collected at the experimental slaughterhouse of the INRAE – Centre Auvergne-Rhone-Alpes at the end of the fattening period. On slaughter day, bulls were fed 2.5 kg of their respective concentrates, 3 h before slaughtering. This feeding protocol was designed to ensure that all animals consumed an identical meal size simultaneously, with no variations in quantity or time taken, mitigating the effect associated with intake levels on the rumen microbiome, as reported by Li et al. (2020a) and for sampling at the peak of rumen microbial activity (Shaani et al., 2018).

Rumen samples (approximately 500 g) were collected from well mixed rumen contents. An aliquot of the total rumen contents was immediately transferred into 15 mL Falcon tubes (5 mL) and snap frozen in liquid nitrogen for RNA studies. For DNA studies, samples were filtered through a polyester monofilament fabric (250 μm mesh aperture), then 1 mL of the filtrate was transferred into 2 mL screw-capped tubes and centrifuged at 14,000 × *g* at 4 °C for 10 min. The supernatant was discarded and the pellet was snap frozen in liquid nitrogen. The samples for RNA and DNA were then stored at −80 °C until analysis. For VFA analysis, 0.8 mL of rumen liquid was aliquoted in 0.5 mL of a 0.5 mol/L HCl solution containing 2% (wt/vol) metaphosphoric acid and 0.4% (wt/vol) crotonic acid. Samples were kept at 4 °C for 2 h before being centrifuged at 16,500 × *g* for 10 min at 4 °C and the supernatant was stored at −20 °C before VFA analysis by GC (Morgavi et al., 2013) using PerkinElmer Clarus 580 + FID equipment (PerkinElmer, Inc, MA, USA).

Rumen pH was continuously monitored throughout the experiment in the 32 extreme RFI bulls using a wireless sensor (eCow, Exeter, UK), as described by Coppa et al. (2023). For the purposes of this study, only the pH values corresponding to 3 h post-feeding were considered in the analysis to ensure comparability with other rumen fermentation variables.

### 2.4 DNA extraction and metataxonomic analysis

Microbial DNA was extracted following the protocol of Yu and Morrison. (2004). Briefly, the rumen fluid pellet was thawed by adding 1 mL of lysis buffer (50 mmol/L Tris–HCl, pH 8, 50 mmol/L EDTA, 4% SDS, 500 mmol/L NaCl), followed by bead beating step at 5,600 round per minute for 3 × 60 s with 5 s pause between cycles on a Precellys 24 machine (Bertin Instruments, Ozyme, Montigny-leBretonneux, France). Subsequent to the cell lysis step, DNA isolation and purification were conducted according to the QIAmp DNA Stool Mini Kit (Qiagen France S.A.S, Les Ulis, France). Genomic DNA (gDNA) was quantified using a Nanodrop Lite spectrophotometer (Thermo Fisher Scientific, Wilmington, USA) and its integrity was checked using the FlashGel system (Lonza, Rockland, Inc., France). Samples of rumen gDNA were sent to ADNID of QUALTECH Group (Montferrier sur Lez, France) for DNA library preparation targeting the 16S rRNA gene (V3–V4) with barcoded primers 341F (5′-CCTAYGGGRBGCASCAG-3′) and 806R (5′-GGACTACNNGGGTATCTAAT-3′); amplicons (466 bp) were sequenced on an Illumina MiSeq platform using a MiSeq reagent kit version 2.

Downstream analysis of 16S rRNA sequences up to the generation of the amplicon sequence variant (ASV) table was conducted using QIIME 2 v.2024.5 (http://qiime2.org/) (Bolyen et al., 2019). The DADA2 method implemented in QIIME 2 v. 2024.5 was applied to model and correct errors introduced during Illumina amplicon sequencing and ASV clustering (Callahan et al., 2016). Taxonomic classification of sequences was assigned using the pretrained classifier Greengenes2 database (https://ftp.microbio.me/greengenes_release/) (McDonald et al., 2023). Tables of ASV, taxonomy, rooted tree, and metadata were merged into a phyloseq object using the phyloseq R package (McMurdie and Holmes, 2013) in R software (v4.4.1; R Core Team, 2024). Two samples (belonging to GS-2019-low RFI and CS-2019-high RFI) were excluded from the analysis. One sample was lost while the other was excluded after quality control and filtering steps due to the low number of reads. To avoid ASV with a small mean and a large coefficient of variation, we removed ASV that did not appear more than three times in at least 25% of the samples in each experimental condition (Diet × Year × RFI; *n* = 4 animals by experimental condition).

### 2.5 RNA extraction and metatranscriptomics analysis

Microbial RNA was extracted using the RNeasy Plus Mini Kit (Qiagen), as previously described (Popova et al., 2017). Total RNA was quantified using a Nanodrop Lite spectrophotometer Fisher Scientific, Wilmington, DE, USA) and its quality was checked on a BioAnalyzer 2100 (Agilent Technologies, Inc., Paris, France). DNA traces were removed with the Invitrogen Turbo DNA-free Kit (Thermo Fisher Scientific SAS, Strasbourg, France) and RNA was purified and concentrated using the RNA Clean & Concentrator kit (Zymo Research, France). Only samples with an RNA integrity number (RIN) ≥ 7.0 were used to generate metatranscriptome libraries. Rumen samples of total RNA were sent to the Genomic Service Center of Genewiz (Leipzig, Germany) for paired-end (2 × 150 bp) sequencing on an Illumina NovaSeq platform. Metatranscriptomics data were analyzed using the open-source MetaTrans pipeline (Martinez et al., 2016). Briefly, the raw paired-end reads were subjected to quality control, such as N content per base (<5%), read length (minimum 150 bp), and quality score per sequence (average quality > 27) using the FastQC tool (Andrews, 2010) and the Kraken pipeline (Wood and Salzberg, 2014). The SortMeRNA tool (Kopylova et al., 2012) was used to separate rRNA/tRNA data from non-rRNA/tRNA reads, the latter being potential mRNAs.

The FragGeneScan tool (Rho et al., 2010) was used with the non-rRNA/tRNA to predict potential genes for functional analysis. The predicted genes were subjected to clustering using CD-HIT v4.6 (Fu et al., 2012) with an identity threshold of ≥95% and gene overlap of ≥90%. For gene annotation, we used a bovine rumen microbial gene catalog (Li et al., 2020b) and the SOAP2 tool (Li et al., 2009). The Kyoto Encyclopedia of Genes and Genomes (KEGG) and evolutionary genealogy of genes of non-supervised orthologous groups (eggNOG) databases were used for annotation. The raw 16S rRNA gene and RNAseq data are available in the ENA project repository, PRJEB70895.

### 2.6 Microbial analysis by qPCR

Copies of the 16S rRNA gene for bacteria and the methyl coenzyme-M reductase (*mcrA*) gene for methanogens were quantified using real-time PCR (qPCR) in a StepOnePlus system (ThermoFisher Scientific, USA). The qPCR quantification was performed in triplicate and the amplification reaction contained 2 μL of gDNA (20 ng/μL), 0.5 μmol/L of each primer, 10 μL of 2 × ONEGreen FAST qPCR Premix (OZYME, France), 0.4 μL of 50 × ROX I and DNA/RNA free water adjusted to final volume of 20 μL. Fragment amplification consisted of an initial activation cycle at 95 °C for 3 min, followed by 40 cycles at 95 °C for 15 s for denaturation and 58 °C for 60 s for hybridization. The primers selected for bacteria were 520F (5′-AGCAGCCGCGGGGTAAT-3′) and 799R (5′-CAGGGTATCTAATCCTGTT-3′) (Edwards et al., 2007) and for methanogens were qmcrAF (5′-TTCGGTGGTGGATCDCDCARAGRG-3′) and qmcrAR (5′-GBARGTCGWAWCCGTAGAGAATCC-3′) (Denman et al., 2007).

Absolute quantification involved the construction of regression curves of the standard with already known concentrations prepared with gDNA from *Methanobrevibacter ruminantium* DSM 1093 and *Prevotella bryantii* DSM 11,371, as described (Popova et al., 2011). For each reaction, the linear regression values of the standard curve were within normal limits (*R*^2^ = 0.99, slope = −3.2 to −3.4 and efficiency = 95% to 100%). The results were expressed as log_10_ gene copies in 1 mL of rumen liquid or in total rumen based on the liquid rumen digesta weight (LRDW). The weight of the total ruminal digesta and the solid and liquid fractions was measured individually for each animal, as previously described (Coppa et al., 2023; Guarnido-Lopez et al., 2022).

### 2.7 Statistical analysis

All statistical analyses and plotting were performed using R Statistical Software (v4.4.1; R Core Team, 2024). A general linear model was used to test the effects of the diets (CS vs. GS), Year (2019 vs. 2020), extreme RFI phenotypes (low RFI vs. high RFI), and their interactions (Diet, Year, RFI, Diet × Year, Diet × RFI, Year × RFI, and Diet × Year × RFI) on pH, fermentation profiles, rumen digesta fractions and the absolute quantification of total bacteria and methanogens. As the Diet × Year × RFI interaction was non-significant for all variables, we excluded it from the final model. The final model was the following:

$$Y_{ijkl}=\mu +\alpha_{j}+\beta_{k}+\gamma_{l}+\alpha_{j}\times \beta_{k}+\alpha_{j}\times \gamma_{l}+\beta_{k}\times \gamma_{l}+{ɛ}_{ijkl}\text{,}$$

where *Y*_*ijkl*_ is the dependent variable for animal *i*, receiving diet *j*, in year *k* and belonging to RFI group *l*; μ is the general mean; *α*_*j*_ is the fixed effect of the type of diet used (*j* = 1, 2), *β*_*k*_ is the fixed effect of the year (*k* = 1, 2), γ_*l*_ is the fixed effect of the RFIs (*l* = 1, 2), α_*j*_ × β_*k*_ is the interaction Diet × Year, α_*j*_ × γ_*l*_ is the interaction Diet × RFI group, *β*_*k*_ × *γ*_*l*_ is the interaction Year × RFI group and ɛ_*ijkl*_ is the residual term.

The mean values in Tables for fermentation profiles, rumen digesta fractions and the absolute qPCR quantification are presented as least-square means with pooled SEM values. To simplify the presentation of the results in the tables, only the effects of Diet, Year, RFI and the Diet × RFI interaction are shown. Tables with all interaction terms are available in the additional information file stored in the open database (https://doi.org/10.57745/5AHA3Q). Effects were declared significant when *P* < 0.05, and a trend was considered when 0.05 ≤ *P* ≤ 0.10.

The statistical workflow for analyzing metataxonomics and metatranscriptomics data involved several steps. First, we tested the main effects (diet, year, and RFI effects) on the whole dataset. We then divided the data into two separate subsets based on diet, and analyzed the RFI effect within each diet to control the diet effect, which potentially masked the true RFI effect. Samples were rarefied at 7641 reads and then alpha and beta diversities were calculated using the Microbiome (v1.27.0) and Phyloseq (v1.49.0) R packages (McMurdie and Holmes, 2013). The alpha diversity indices included observed species, Shannon diversity and phylogenetic diversity (PD) at the ASV level. The beta dispersion test was performed to check the ASV dispersion between the experimental groups using the betadisper function in the vegan (v2.6-7) R library (Oksanen et al., 2015). Beta diversity was performed using the Bray–Curtis dissimilarity metric at ASV level followed by the Adonis2 test of permutational multivariate analysis of variance (PERMANOVA) to test the significance of clustering between experimental groups. Distance-based redundancy analysis (dbRDA) was performed and plotted using the MicroViz (v0.12.4) (Barnett et al., 2021) and vegan R libraries, at the taxonomic family level with centered log-ratio transformation (CLR) of the counts, and the constrained variables were Diet, Year and RFI groups. The ordination plot of ASV within each diet was plotted using the non-metric multidimensional scaling (NMDS) analysis.

Microbiota differential abundance analysis (DAA) was performed using Microbiome Multivariable Associations with Linear Models (MaAslin2 v1.18.0) (Mallick et al., 2021) in R. The analysis followed the approach described above. We first tested the main effects on the whole dataset, but to simplify the tables, we have only showed the results for the diet and RFI effects. The analysis within each diet included the effect of Year, RFI, and the Year × RFI interaction, but again to simplify the results presentation, we have only displayed the results for the RFI effect. All QIIME2 output data, and DAA results and their significance values are available in the additional information file stored in the open database (https://doi.org/10.57745/5AHA3Q). The command setup included data normalization (CLR transformation) and adjustment of prevalence parameters (≥10%), minimum abundance (≥0.1%) and a maximum significance value of adjusted *P*-value (*q* < 0.10).

For metatranscriptomics, data were filtered before downstream analysis as for metataxonomic analysis. The pretreatment removed KEGG that did not appear more than three times in at least 25% of the samples in each experimental condition. To provide an overview of the data, KEGG Orthology (KO) related genes were categorized into observations of functions, KEGG metabolism and Clusters of Orthologous Genes (COG) functions, using the Shotgun Data Profiling of MicrobiomeAnalyst web platform (Chong et al., 2020) (https://www.microbiomeanalyst.ca/). Counts were then normalized to counts per million reads, and differences in abundances between experimental groups were analyzed using a multivariate linear model (MTX model v1.2.5) (Zhang et al., 2021a). Briefly, the analysis setup did not include data normalization and transformation, but it was adjusted for prevalence parameters (≥10%), minimum abundance (≥0.1%), and a maximum significance value of adjusted *P*-value (*q* < 0.10).

Furthermore, differentially expressed KEGG (deKEGG) between experimental groups was determined using the DESeq2 (v1.44.0) R package (Love et al., 2014) with default parameters. DESeq2 uses un-normalized count data as input, and it internally corrects for library size. The design formula used for the whole dataset was “KEGG = Diet + RFI” and for each diet, it was “KEGG = RFI”. In addition, shrinkage estimators of effect size (lfcSE) were fitted using the lfcShrink function in DESeq2 with the apeglm method (Zhu et al., 2019). Differences were considered significant after the adjusted *P*-value (*q* < 0.10) using the Benjamini Hochberg FDR (False Discovery Rate) procedure unless otherwise indicated in the table or figure. After differential expression analysis, enrichment analysis of the deKEGG was performed using the ExpressAnalyst web platform (https://www.expressanalyst.ca/) (Ewald et al., 2023) using overrepresentation analysis (Sherman et al., 2022) and the significant functional categories were visualized using a ridgeline chart.

## 3 Results

### 3.1 Fermentation profiles and microbial quantification

The rumen pH did not differ between diets, or between years, but tended to be lower in the most efficient bulls than in the least efficient bulls (*P* = 0.097). The total VFA concentration in the rumen did not differ between the extreme RFI phenotypes or between years, and it tended to be higher (*P* = 0.071) in the CS diet than in the GS diet (Table 2). The VFA profile and the acetate to propionate (A:P) ratio did not differ between the extreme RFI phenotypes. However, diet had a significant effect on the VFA profiles, with the CS diet inducing a higher concentration of acetate and minor VFA. When the VFA profile was expressed as a percentage of total VFA, propionate (%) was higher in the GS than in the CS diet. The A/P ratio was higher in the CS diet. Additionally, the year factor had a significant effect on iso-butyrate, valerate, and iso-valerate (*P* < 0.05). However, no interaction effect for Diet × Year, Diet × RFI, or Year × RFI (*P* > 0.05) was observed.

**Table 2:** Rumen fermentation profiles of extreme RFI Charolais young bulls fed either a CS or GS diet.^1^

Item	GS		CS		SEM	*P*-value			
	Low RFI	High RFI	Low RFI	High RFI		Diet	Year	RFI	Diet × RFI
pH	6.4	6.5	6.3	6.5	0.08	0.706	0.142	0.097	0.412
Total VFA, mmol/L	148	148	170	166	13.6	0.071	0.151	0.788	0.841
Acetate, mmol/L	102	103	119	116	9.5	0.048	0.239	0.931	0.797
Propionate, mmol/L	26.2	25.8	26.6	25.6	2.65	0.976	0.143	0.733	0.865
Butyrate, mmol/L	14.1	13.6	15.5	14.4	1.82	0.426	0.527	0.574	0.836
Iso-butyrate, mmol/L	1.47	1.41	1.84	1.79	0.165	0.005	0.001	0.677	0.976
Iso-valerate, mmol/L	1.66	1.49	3.10	2.82	0.340	0.001	0.001	0.391	0.834
Valerate, mmol/L	2.03	1.69	2.74	2.51	0.324	0.004	0.004	0.265	0.822
Caproate, mmol/L	0.50	0.41	1.26	1.09	0.137	0.001	0.197	0.214	0.748
Acetate, %	69.2	70.0	69.9	70.8	1.41	0.526	0.170	0.503	0.970
Propionate, %	17.7	17.6	15.6	15.5	0.64	0.001	0.177	0.819	0.994
Butyrate, %	9.4	9.0	9.2	8.8	0.78	0.722	0.581	0.535	0.950
Iso-butyrate, %	1.0	1.0	1.1	1.1	0.10	0.132	0.022	0.823	0.847
Iso-valerate, %	1.1	1.0	1.8	1.7	0.19	0.001	0.001	0.635	0.763
Valerate, %	1.3	1.1	1.6	1.5	0.16	0.011	0.009	0.293	0.728
Caproate, %	0.3	0.3	0.7	0.7	0.07	0.001	0.848	0.252	0.915
A:P ratio	4.0	4.0	4.5	4.6	0.24	0.005	0.167	0.750	0.938

RFI = residual feed intake; CS = corn silage; GS = grass silage; VFA = volatile fatty acids; A:P = acetate to propionate ratio; SEM = standard error of the mean.

1 Each value represents the LS means per experimental group (GS–Low RFI, *n* = 8; GS–High RFI, *n* = 8; CS–Low RFI, *n* = 8; CS–High RFI, *n* = 7).

The weight of the mixed rumen digesta was lighter in the most efficient compared to the least efficient bulls (*P* = 0.027), and the weight of the liquid (*P* = 0.054) and dried (*P* = 0.079) fractions tended to be lighter in the most efficient bulls (Table 3). The diet had no effect on rumen digesta fractions (*P* > 0.05). However, the year had an effect on the weights of mixed and liquid rumen digesta (*P* < 0.01), and a trend was observed for dried rumen digesta (*P* = 0.072). In contrast, the weight of rumen digesta fractions (i.e., mixed, dried, and liquid) showed no Diet × RFI interaction (*P* > 0.05).

**Table 3:** Rumen digesta and population of bacteria and methanogens of extreme RFI Charolais young bulls fed either a CS or GS diet.^1^

Item	GS		CS		SEM	*P*-value			
	Low RFI	High RFI	Low RFI	High RFI		Diet	Year	RFI	Diet × RFI
**Rumen digesta weight, kg**									
Mixed	38.8	43.0	37.9	43.6	2.80	0.992	0.001	0.027	0.711
Dried	5.1	5.5	5.3	6.2	0.45	0.285	0.072	0.079	0.524
Liquid	33.7	37.6	32.5	35.3	2.14	0.268	0.004	0.054	0.756
**Absolute quantification**^2^									
Total bacteria	10.30	10.27	10.31	10.35	0.090	0.569	0.001	0.955	0.609
Total bacteria (LRDW)	14.83	14.83	14.82	14.89	0.085	0.796	0.001	0.576	0.582
Methanogens	9.07	9.13	8.86	8.96	0.092	0.011	0.004	0.310	0.500
Methanogens (LRDW)	13.59	13.69	13.36	13.53	0.096	0.011	0.001	0.101	0.842

RFI = residual feed intake; CS = corn silage; GS = grass silage; LRDW = liquid rumen digesta weight; SEM = standard error of the mean.

1 Each value represents the LS means per experimental group (GS = Low RFI, *n* = 8; GS = High RFI, *n* = 8; CS = Low RFI, *n* = 8; CS = High RFI, *n* = 7).

2 The results were expressed as log_10_ gene copies in 1 mL of rumen liquid or in total rumen based on the LRDW.

Total bacterial and methanogen populations did not differ between the extreme RFI phenotypes (*P* > 0.05). When results were expressed as copies per LRDW, the tendency was for fewer methanogens in the most efficient bulls than in the least efficient bulls (*P* = 0.101) (Table 3). Total bacteria did not differ between dietary treatments, but methanogens numbers were higher in the GS diet than in the CS (*P* < 0.05) diet. Furthermore, bacterial and methanogen populations were more abundant in 2020 than in 2019 (*P* < 0.05). Total bacterial and methanogen populations showed no Diet × RFI interaction (*P* > 0.05).

### 3.2 Diet was the main structuring factor of the rumen microbiota

**Fig. 1:**
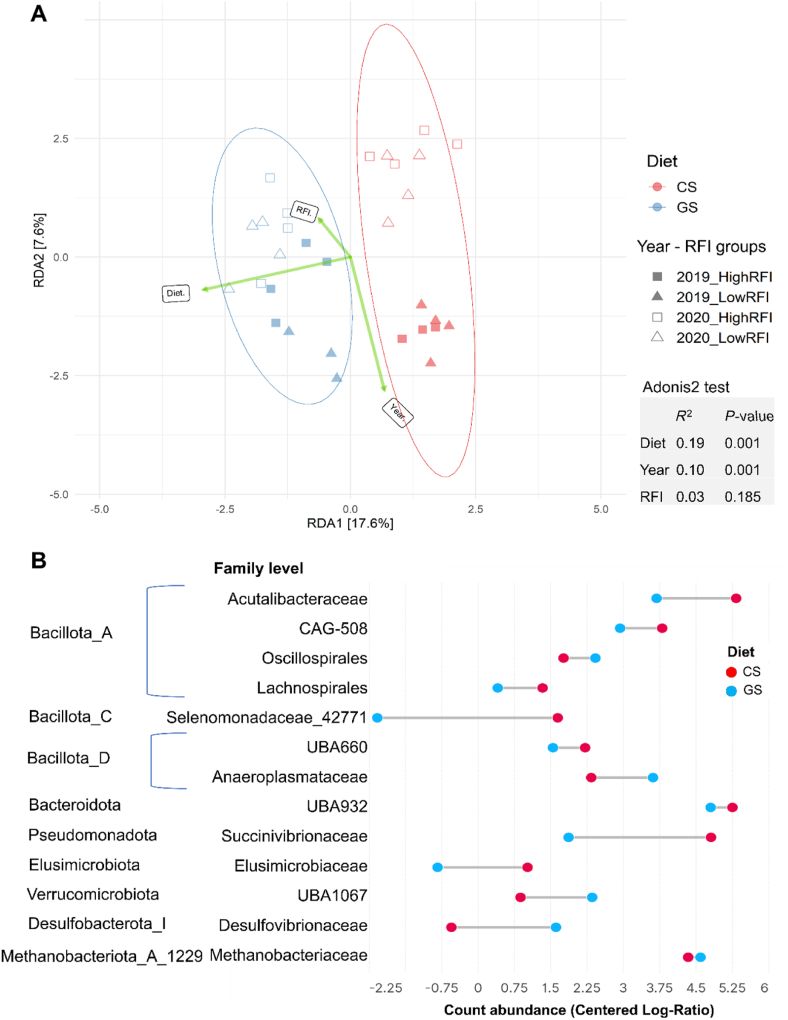
Microbial community structure and abundance in relation to diet and residual feed intake (RFI). The study was conducted for two consecutive years: 2019 and 2020. (A) Redundancy analysis where “Diet”, “Year”, and “RFI” (green arrows) were used as constrained variables. Samples were plotted along the first two component axes. (B) Identification of rumen discriminant microbes at the family level in the most (low RFI) or the least efficient (high RFI) young Charolais bulls fed either grass silage (GS) or corn silage (CS) diets. The significantly discriminant taxa (*q* < 0.1) were computed based on centered log-ratio (CLR) normalization and Benjamini-Hochberg false discovery rate (FDR) procedure at family or genus level.

Analysis of the whole dataset showed that diet was the main driver modulating the rumen microbial community (Adonis2 Test, *R*^2^ = 0.19, *P* < 0.001) (Fig. 1A) with no differences found between the extreme RFI phenotypes (Adonis2 Test, *R*^2^ = 0.03, *P* = 0.185). Alpha diversity indices did not differ between diets or RFI groups (*P* > 0.05) (Table S1), but there were differences between years, with a higher Shannon index in 2019 than in 2020 (*P* < 0.05).

As expected, a large number of taxa at the family and genus levels was discriminant between diets (Fig. 1B and Fig. S1A). In particular, bulls fed the GS diet had a higher proportion of microbes belonging to the families Anaeroplasmataceae (Bacillota_D), Oscillospirales (Bacillota_A), UBA1067 (Verrucomicrobiota), Desulfovibrionaceae (Desulfobacterota_I) and Methanobacteriaceae (Methanobacteriota_A_1229), than those fed the CS diet (*q* < 0.10). On the other hand, microbes belonging to Acutalibacteraceae (Bacillota_A), Selenomonadaceae_42771 (Bacillota_C), UBA932 (Bacteroidota), and Succinivibrionaceae (Pseudomonadota) were more abundant in the CS diet than in the GS diet (*q* < 0.10). In contrast, only a few taxa were found to be discriminant between the extreme RFI phenotypes (Fig. S1B). Sugar-fermenting microbes, e.g., *Succiniclasticum* and *Saccharofermentans* were more abundant in the least efficient bulls than in the most efficient bulls (*q* < 0.10).

### 3.3 Rumen microbiota is linked to extreme RFI phenotypes within each diet

**Fig. 2:**
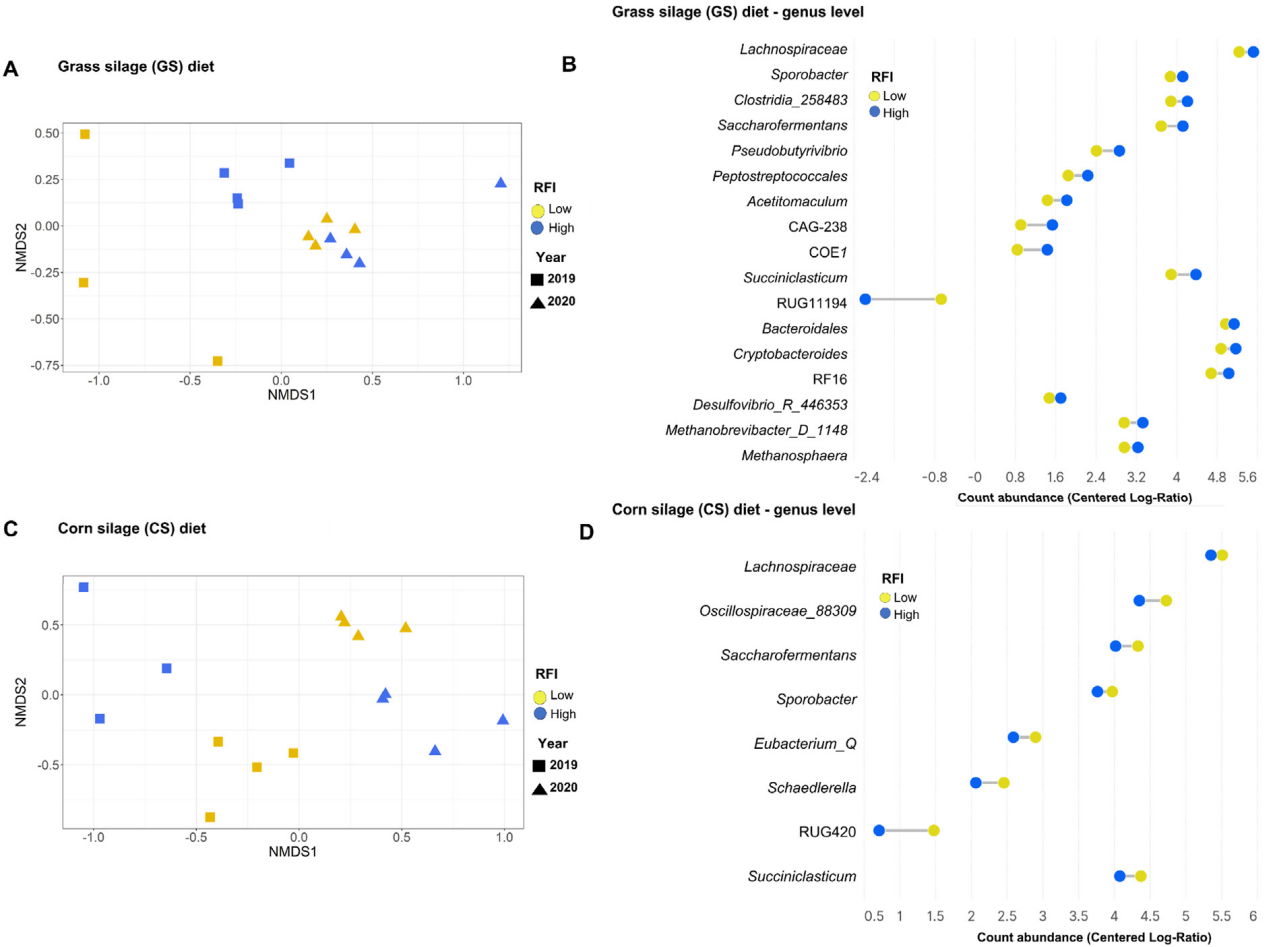
Microbial community structure and abundance in the most (low RFI) or the least efficient (high RFI) young Charolais bulls fed either a grass silage (GS) diet (A and B) or a corn silage (CS) diet (C and D). The effects of experimental year, residual feed intake (RFI) and year × RFI on rumen microbial community structure (A and C) were determined using the Adonis2 test. The non-metric multidimensional scaling (NMDS) plots derived from Bray Curtis dissimilarity analysis between bulls. Samples were plotted along the first two component axes. For B and D significantly discriminant taxa (*q* < 0.1) were computed based on centered log-ratio (CLR) normalization and Benjamini-Hochberg false discovery rate (FDR) procedure at the genus level. RFI = residual feed intake.

Rumen microbial communities differed between the extreme RFI phenotypes within each diet. In the GS diet, RFI modulated the rumen microbial community (Adonis2 Test, *R*^2^ = 0.09, *P* = 0.043) (Fig. 2A) with no effect on alpha diversity (Table S1). The year also had an effect on beta and alpha diversities for all indices (species observed, Shannon and PD), which were higher in 2019 than in 2020 (*P* < 0.05; Year: *R*^2^ = 0.20, *P* < 0.001). Fifteen bacterial and two methanogens’ genera were identified as discriminants between the extreme RFI phenotypes. Their relative abundance accounted for 30.5% of the total in the most (low RFI) and 35.2% in the least (high RFI) efficient bulls (Fig. 2B). All the other discriminant genera, with the exception of the low abundant RUG11194, were more abundant in the least efficient bulls compared to the most efficient bulls (*q* < 0.10). In the CS diet, the microbial community did not differ between extreme RFI phenotypes (Adonis2 Test, *R*^2^ = 0.07, *P* = 0.149) but it did differ between years (Adonis2 Test, *R*^2^ = 0.25, *P* = 0.001) (Fig. 2C). Alpha diversity indices did not differ between extreme RFI phenotypes or years (*P* > 0.05) (Table S1). Notwithstanding the absence of beta diversity, eight discriminant bacterial genera were detected, accounting for 16.3% of the total in the most efficient bulls and 15% in the least efficient bulls (Fig. 2D). All these discriminant bacterial genera were more abundant in the most efficient bulls (*q* < 0.10).

Additional analyses were conducted within each year to assess the effect of diet and RFI, and to verify whether any microbial variables were masked by the year effect. Given that the results were comparable to those obtained with the whole dataset, only the results described above are presented here.

### 3.4 Diet is also the main driver of the rumen microbial functional profile

**Fig. 3:**
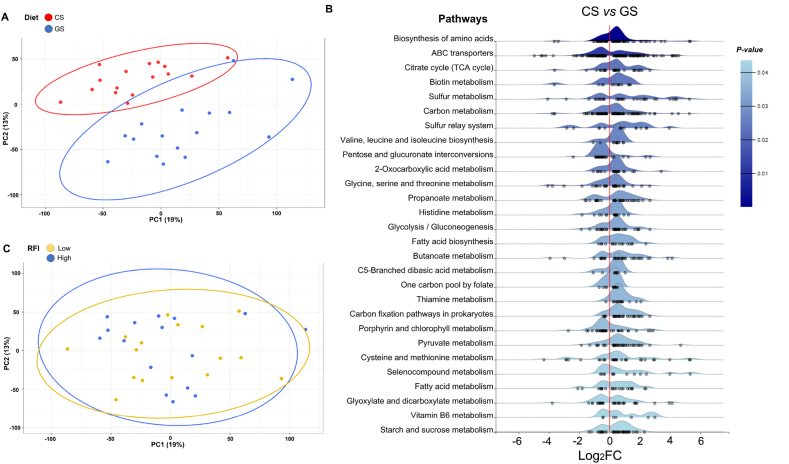
Rumen functional microbial profiling in the most (low RFI) or the least efficient (high RFI) young Charolais bulls fed either corn silage (CS) or grass silage (GS) diets. (A) Principal component analysis (PCA) of the diet effect on gene transcripts detected in the rumen fluid. (B) Ridgeline plot of 29 enriched pathways comparing the CS vs GS diet, and (C) PCA of the residual feed intake (RFI) effect on gene transcripts detected in the rumen fluid. Samples were plotted along the first two component axes after centered log-ratio (CLR) normalization of counts. RFI = residual feed intake.

Similar to metataxonomics, metatranscriptomics revealed a strong impact of the diet on the functional profiles of the rumen microbiome (Fig. 3A). Out of the total 10,535 KEGG detected, 3,174 were differentially expressed between diets, confirming the influence of the diet on the functional profile of the microbiome. There were 28 enriched metabolic pathways (Fig. 3B), particularly those related to the biosynthesis of amino acids (valine, leucine and isoleucine biosynthesis, and glycine, serine and threonine metabolism), carbohydrate metabolism (e.g., starch and sucrose metabolism, pyruvate metabolism, and propanoate metabolism), and fatty acid metabolism. Notably, the vast majority of deKEGG related to the biosynthesis of amino acids, biotin metabolism, carbon metabolism, fatty acid biosynthesis and metabolism were overexpressed in the CS diet compared to the GS (*q* < 0.10) diet. On the other hand, ABC transporter, pentose and glucuronate interconversions, porphyrin and chlorophyll metabolism, selenocompound metabolism and glyoxylate and dicarboxylate metabolism were overexpressed in GS compared to the CS diet. Although methane metabolism (from carbon metabolism pathways) displayed similar patterns between the diets, methylamine/dimethylamine/trimethylamine (K03388, K03389, K14126, and K14128) and methanol (K04480, K14080 and K14081), which are substrates used in the production of methane via the hydrogen-dependent methylotrophic pathway, were overexpressed in the GS diet.

Similarly, for the general functional categories of KEGG, we found a higher abundance of glycan biosynthesis and metabolism, nucleotide metabolism, and biosynthesis of other secondary metabolites in the GS than in the CS diet (*q* < 0.10) (Table S2). In contrast, in the COG categories, a higher abundance of defense mechanisms, and energy production and conversion were observed in the CS diet (*q* < 0.05). No differences were found between diets for the other functional categories of KEGG and COGs (*q* > 0.05).

In the PCA ordination plot for the whole dataset (Fig. 3C) and for each diet (Figs. S2A and S2B), there was no clustering between extreme RFI phenotypes, and there were no significant differences for any general functional categories of KEGG and COG (*q* > 0.10) (Table S3). Furthermore, when examining the RFI effect for each diet, no differences were found between the extreme RFI phenotypes for any general KEGG functional categories (*q* > 0.10) (Table 4).

**Table 4:** Microbial functional category abundances (means of counts per million reads ± SD) of extreme RFI Charolais young bulls fed either a CS or GS diet.^1^

KEGG functional category	Low RFI	High RFI	*q*-value^2^
**GS diet**			
Carbohydrate metabolism	288,000 ± 7,223	305,000 ± 7,486	0.290
Energy metabolism	232,451 ± 9,677	235,930 ± 3,473	0.796
Amino acid metabolism	175,890 ± 8,207	164,178 ± 7,419	0.583
Nucleotide metabolism	88,510 ± 3,129	86,049 ± 2,185	0.611
Metabolism of cofactors and vitamins	56,369 ± 5,533	51,582 ± 2,327	0.583
Lipid metabolism	38,484 ± 1,310	39,770 ± 1,073	0.617
Metabolism of other amino acids	32,154 ± 1,178	33,216 ± 1,253	0.634
Xenobiotic biodegradation and metabolism	25,523 ± 5,013	20,513 ± 497	0.583
Glycan biosynthesis and metabolism	23,809 ± 1,351	22,658 ± 1,155	0.611
Biosynthesis of other secondary metabolites	21,093 ± 724	23,460 ± 1,249	0.331
Metabolism of terpenoids and polyketides	17,038 ± 744	16,942 ± 597	0.932
**CS diet**			
Carbohydrate metabolism	304,700 ± 9,051	305,000 ± 6,793	0.978
Energy metabolism	247,000 ± 3,148	246,000 ± 6,323	0.978
Amino acid metabolism	170,000 ± 11,329	166,000 ± 8,602	0.978
Nucleotide metabolism	78,706 ± 2,536	78,586 ± 3,289	0.978
Metabolism of cofactors and vitamins	54,554 ± 1,692	56,593 ± 1,919	0.978
Lipid metabolism	36,972 ± 1,600	37,174 ± 1,903	0.978
Metabolism of other amino acids	31,235 ± 1,185	31,430 ± 819	0.978
Xenobiotic biodegradation and metabolism	21,190 ± 772	21,752 ± 791	0.978
Glycan biosynthesis and metabolism	19,884 ± 875	20,138 ± 1,090	0.978
Biosynthesis of other secondary metabolites	19,760 ± 1,256	19,436 ± 867	0.978
Metabolism of terpenoids and polyketides	16,274 ± 640	17,230 ± 1,080	0.978

RFI = residual feed intake; CS = corn silage; GS = grass silage; SD = standard deviation.

1 Each value represents the mean of counts per million reads per experimental group (GS–Low RFI, *n* = 8; GS–High RFI, *n* = 8; CS–Low RFI, *n* = 8; CS–High RFI, *n* = 8).

2 The *q*-value was obtained after false discovery rate (FDR) correction using the Benjamini-Hochberg procedure.

In the CS and GS diets, we found 282 and 253 deKEGG, respectively between the extreme RFI phenotypes (*q* < 0.10), both accounting for less than 0.1% of the total reads. Because these deKEGG had lower abundance counts and were not related to any relevant microbial metabolic pathways, we did not perform further analyses.

## 4 Discussion

This RFI study has the particularity of using two different diets for fattening beef cattle that were compared in parallel. In addition, bulls were sampled after feeding an equal meal size, ensuring a homogeneous comparison between diets and RFI rankings to test the hypothesis that distinctive taxonomic and functional rumen microbiome characteristics would be observed in high and low RFI bulls regardless of diet. Sampling time relative to feeding is an important factor to consider because fermentation activity and dynamics of rumen microbes, both in absolute and relative abundances are conditioned by the arrival of ingested feed into the reticulo-rumen (Sun and Gibbs, 2012). We considered that differences between extreme RFI phenotypes may become more evident 3 h after feeding, as this period coincides with a peak in microbial activity (van Lingen et al., 2017; Shaani et al., 2018).

Microbial beta diversity was strongly influenced by diet, and this effect is likely to have masked changes associated with RFI that are expected to be more subtle. Yet, considering the whole dataset, we identified *Succiniclasticum*, *Saccharofermentans*, *Clostridia_258483* and CAG-238 as discriminant bacteria between extreme RFI phenotypes regardless of the diet, which partially confirms our hypothesis. However, the direction of the association, negative or positive, with RFI was diet dependent, for example, in bulls fed the GS diet, these microbes were positively associated with the least efficient bulls. In contrast, in the CS diet, only *Saccharofermentans* and *Succiniclasticum* were positively associated with the most efficient bulls, suggesting an interaction between diet and RFI. These microbes are sugar fermenters involved in energy utilization in the rumen and are enriched in energy-dense diets (Hu et al., 2020). *Saccharofermentans* produce fumarate, lactate and acetate as end products of starch fermentation (Chen et al., 2010). Whereas, *Succiniclasticum* utilizes succinate, converting it to propionate (van Gylswyk, 1995). These microbes have also been positively associated with efficient ruminants fed high-concentrate diets (Auffret et al., 2020; Zhang et al., 2021a, Zhang et al., 2021b). The starch content in the CS diet is lower than that in the diets reported by Auffret et al. (2020) (33% vs. >40%) but it is noted that samples in this study were taken 3 h post-feeding a concentrate meal, which may have favored the growth of these species. Thus, these results indicate that some microbes are distinctive between the extreme RFI phenotypes. However, we cannot explain why the association was positive or negative depending on the diet. Further studies would be needed to clarify this point.

Our results, in Charolais bulls fed the GS diet showed significant differences in microbial structure between the extreme RFI phenotypes. This finding is in agreement with two reports on Angus heifers fed an equal ratio of concentrate to forage (50: 50) (Clemmons et al., 2022; Liu et al., 2022). However, most previous reports in beef cattle found no significant differences in rumen microbial structure between extreme RFI phenotypes. Host-related factors (breed, sex and age), diet and sampling time relative to feeding may contribute to the inconsistencies reported in the literature (see Table S4). Furthermore, prolonged low rumen pH (< 5.8) has been demonstrated to induce changes in the structure and function of the rumen microbiota (Yang et al., 2022). Nevertheless, in our study, rumen pH (close to 6.5) was much higher than the 5.8 threshold and only slightly lower than the pre-feeding pH of 6.7 reported by Liu et al. (2022). This may indicate that rumen pH relative to the feeding period cannot explain the contrasting results reported previously.

Interestingly, in the CS diet, genera from Bacillota A and C, *Succiniclasticum*, *Saccharofermentans*, *Lachnospiraceae* and *Oscillospiraceae_88309*, were the key microbes associated with improved RFI. These microbes have been reported to have a positive association with the rumen concentration of VFA (van Gylswyk, 1995; Hu et al., 2020; Kaminsky et al., 2023). The association of some of these microbes, mainly *Saccharofermentans* and *Lachnospiraceae*, with the efficient phenotypes in lambs (Zhang et al., 2021a, Zhang et al., 2021b) and beef cattle (Clemmons et al., 2022) fed high-concentrate (>60%) diets has also been reported. The nature of this association may change depending on the diet, as mentioned above. Most published studies have been conducted with high-concentrate diets or diets with a high proportion of corn silage. However, there is insufficient information on diets with a low proportion of concentrate and on diets where the main forage component is not corn silage. This deserves more attention as diets vary according to the age and production stage of ruminants and also according to geographical location.

Overall, these results may indicate microbial changes favoring rumen performance in the most efficient cattle. However, our results for VFA fermentation and metatranscriptomics do not fully support this idea. The reason for this lack of consistency could be related to the effect of the feeding prior to sampling. Instead of increasing the differences between RFI-ranked bulls as expected, giving a meal of the concentrate portion of the diet may have decreased them. Perhaps a single sampling time was not sufficient to detect these possible differences, considering that fermentation kinetics and the rates of VFA production and absorption are influenced by factors such as diet, rumen osmolarity, metabolic hydrogen flows, among others (Dijkstra, 1994; Ungerfeld, 2020). Also, the nature of the rumen samples used in this study, liquid for metataxonomics and whole content for metatranscriptomics, could influence the microbial results, as reported for different rumen digesta fractions (McLoughlin et al., 2023).

In our metatranscriptomic results, the least efficient bulls had a numerically, although not significantly, higher abundance of transcripts in general functional categories, such as metabolism of carbohydrates, energy, and lipids. In contrast, the most efficient bulls had a numerically higher abundance of transcripts for the metabolism of amino acids, nucleotides, cofactors, and vitamins. There are few reports that examine the relationship between microbial functional features and feed efficiency in cattle. These reports, which used metatranscriptomics (Li and Guan, 2017) and metagenomics (Shabat et al., 2016; Xie et al., 2022), revealed that the majority of discriminant metabolic pathways (at the KEGG and CAZyme levels) increased in least efficient cattle. Given the complexity and dynamics of the rumen and its microbes, a better assessment of the relationship between host RFI and changes in microbiota function could be achieved in future studies using time series sampling and additional approaches, e.g., metabolomics and proteomics.

Lower enteric methane emission is another trait that has been associated with efficiency in cattle (Arndt et al., 2022; Roques et al., 2024). Interestingly, in this study, the most efficient bulls tended to have a lower population of methanogens when expressed per LRDW, regardless of the diet. This coincides with a lower relative abundance of *Methanobrevibacter_D_1148* and *Methanosphaera* spp. in the most efficient bulls fed the GS diet. The relationship with *Methanosphera* spp. in the GS diet may be due to the high pectin content in beet pulp, as methanol is a byproduct of pectin hydrolysis and *Methanosphera* utilizes solely methanol to produce methane (Fricke et al., 2006). These findings are in agreement with reports in cattle fed low (Zhou et al., 2009), moderate (Xie et al., 2022) and high concentrate (Shabat et al., 2016) diets. The higher abundance of methanogens in the least efficient bulls is in accord with the greater methane emissions measured on these animals as compared with high RFI bulls (Bes et al., 2022). The greater methanogenic activity could be primarily attributed to higher substrate availability, e.g., formate, dihydrogen, and methyl compounds, resulting from increased and more diverse carbohydrate metabolic activity, as reported in both beef and dairy cattle (Li and Guan, 2017; Shabat et al., 2016; Xie et al., 2022).

Based on the results of this study and existing published information, there is no emerging microbial profile associated with feed efficiency, even when considering only studies on beef cattle of similar age and the rumen microbiome. Given the strong effect of diet on the microbiome, this lack of common results among studies can come from differences in diet composition even for diets seemingly similar as observed in this study for different years (see below).

Diet is the most important factor influencing the rumen microbiome and fermentation parameters, including enteric methane production. Our results attest once more to this accepted view (particularities of the results are discussed in Supplementary Information). What is relevant in this highly controlled study is that differences between the experimental years (2019 and 2020) were also noticeable in the rumen microbiome. The cause of these differences lies in the slight variations in silage quality from one year to another (Guarnido-Lopez et al., 2022). Thus, if relatively small variations in feed components induce manifest differences in the rumen microbiome, it may make changes associated with RFI phenotypes more difficult to detect. This may be one reason why there is no clear pattern of microbial changes or indicator species in the published literature.

Identifying robust and generalizable microbial targets associated with highly efficient phenotypes remains a challenging task. A task that is further complicated by the high heterogeneity of methods for microbial analysis and the fairness of their data. Future studies involving a larger number of animals, the use of different diets and the harmonization of statistical and bioinformatic methods will be necessary to arrive at conclusive results. A meta-analysis approach to existing studies can also be a source of novel information.

## 5 Conclusions

In this study of beef cattle, we found a distinct microbial profile and indicator species between extreme RFI phenotypes across two diets that were not reported before. However, these microbial differences were moderate and there was an interaction with diet, which remained the main modulator of the microbiome. Metataxonomic changes were observed more consistently than functional changes, as no differences were observed in fermentation products or the functional profile of the microbiome between the extreme RFI phenotypes. Confirmation and further assessment of these results should be done in order to use this approach as a predictive and management tool to improve feed efficiency in beef production systems.

## Credit Author Statement

**Abimael Ortiz-Chura:** Writing – original draft, Methodology, Formal analysis. **Karla Fabiola Corral-Jara:** Software, Methodology. **Jeremy Tournayre:** Software, Methodology, Data curation. **Gonzalo Cantalapiedra-Hijar:** Writing – review & editing, Investigation, Funding acquisition, Conceptualization. **Milka Popova:** Writing – review & editing, Supervision, Investigation, Funding acquisition, Conceptualization. **Diego P. Morgavi:** Writing – review & editing, Supervision, Investigation, Funding acquisition, Conceptualization.

## Availability of data and material

Sequence data can be found at https://www.ebi.ac.uk/ena/browser/view/PRJEB70895. Other material and analysis are available at https://doi.org/10.57745/5AHA3Q.

## Declarations of competing interest

We declare that we have no financial and personal relationships with other people or organizations that can inappropriately influence our work, and there is no professional or other personal interest of any nature or kind in any product, service and/or company that could be construed as influencing the content of this paper.
