# Supplementary material for: Rumen microbiota associated with feed efficiency in beef cattle are highly influenced by diet composition
Source: Anim Nutr. 2025 Mar 15;21:378–89. doi: 10.1016/j.aninu.2024.11.027 (PMC12143644; doi:10.1016/j.aninu.2024.11.027)
Supplement: Multimedia component 1 [file mmc1.doc]

### **Diet as a main structural driver of the rumen microbiome**

Bulls fed the GS diet had a higher abundance of methanogens in both absolute and relative terms, along with increased activity in the dissimilatory methylotrophic pathways (methylamine/dimethylamine/trimethylamine and methanol) of methanogenesis compared to the CS diet. These findings align with the higher methane yield (g/kg DMI) reported in these bulls fed the GS diet compared with those fed the CS diet (Bes et al., 2022). The reason for this higher methanogenic activity can be attributed to the high pectin content (approximately 40%) in the beet pulp ingredient (Oosterveld et al., 1996) present in the GS diet. Pectin polysaccharides have varying degrees of methylation, and their hydrolysis in the rumen produces methanol, resulting in increased methanogenesis (Sun et al., 2022). Betaine is another compound found in beet pulp, that is catabolized into trimethylamine by rumen microbes, subsequently serving as a direct substrate for methylotrophic methanogenesis (Mahmood et al., 2020; Patterson and Hespell, 1979). Furthermore, bulls fed the GS diet had a higher abundance of the families, *Lachnospiraceae*, *Bacteroidaceae* and *Oscillospirales,* and they were associated with degradation of fibrous plant material and pectin (Dehority, 1969; Thoetkiattikul et al., 2013). Conversely, bulls fed the CS diet stimulated the growth of bacteria involved in propionate and valerate synthesis, such as some members of the families *Acutalibacteraceae*, *Selenomonadaceae_42771* and *Succinivibrionaceae* (Blackburn and Hungate, 1963; Wang et al., 2020). In this study, although some KEGG related to propionate synthesis, such as starch and sucrose metabolism and pyruvate metabolism were overexpressed in the CS diet compared to the GS diet, differences in the fermentative profile of the three major VFA did not vary between diets. However, the total VFA production tended to be higher in the CS diet than in the GS diet, which correlated with the higher abundance of KEGG related to functional categories such as energy production and conversion, carbohydrate metabolism, and energy metabolism (although the latter two were only numerically higher in the CS diet). The similarity in the fermentation profile for both diets can be attributed to the fact that the bulls received only the concentrated component of the diet before slaughter.





**Fig. S1.** Identification of rumen discriminant microbes at the genus level in the most (low RFI) or the least efficient (high RFI) young Charolais bulls fed either grass silage (GS) or corn silage (CS) diets. (**A**) effect of the diet. (**B**) Effect of residual feed intake (RFI). For both figures, the significantly discriminant taxa (*q* < 0.1) were computed based on centered log-ratio (CLR) normalization and Benjamini-Hochberg false discovery rate (FDR) procedure at genus level.


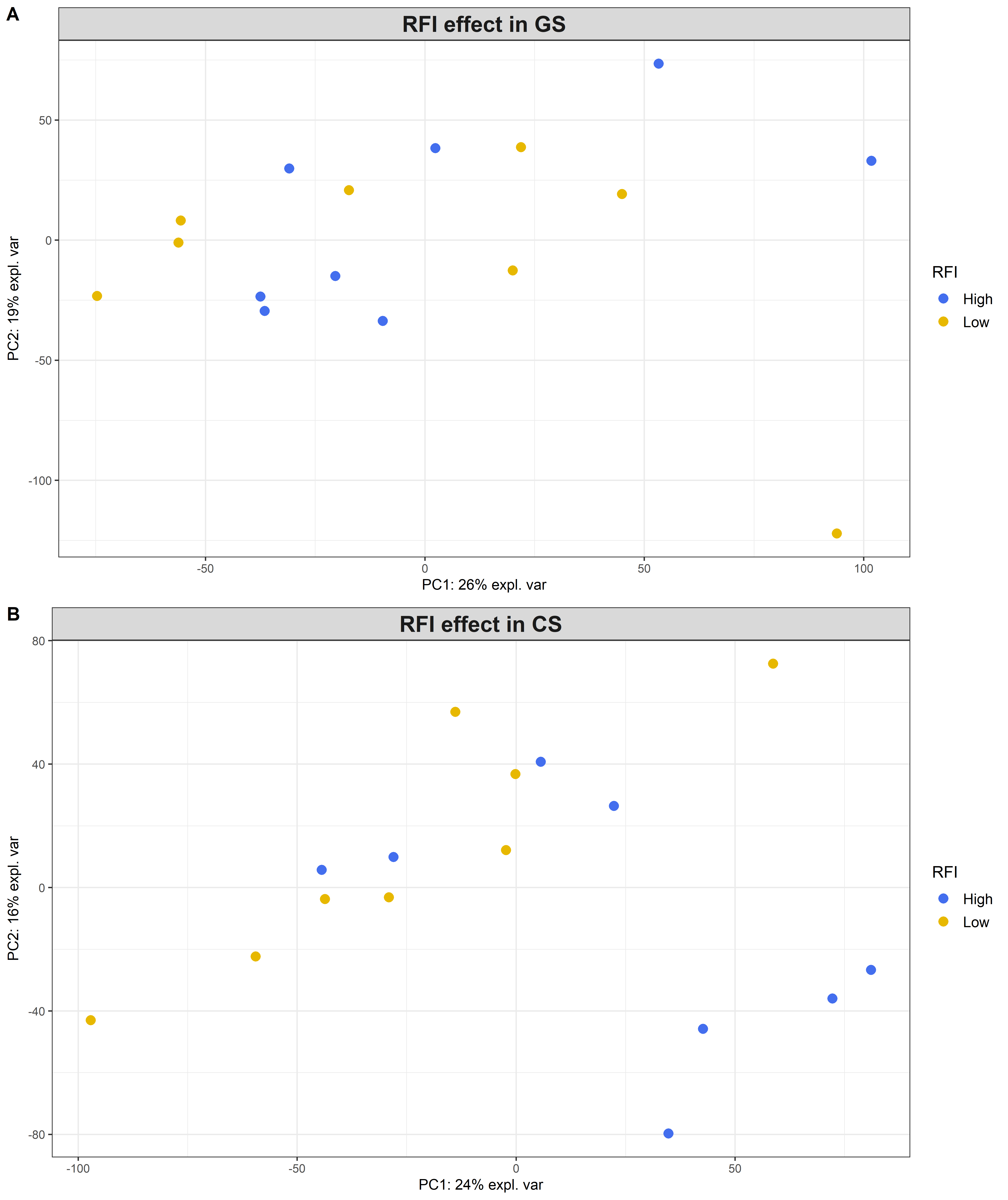


**Fig. S2**. Rumen functional microbial profiling in the most (low RFI) or the least efficient (high RFI) young Charolais bulls fed either grass silage (GS) or corn silage (CS) diets. Principal component analysis (PCA) of the residual feed intake (RFI) effect within the GS (**A**) or CS (**B**) diets on gene transcripts detected in the rumen fluid. Samples were plotted along the first two component axes after centered log-ratio (CLR) normalization of counts.

**Table S1.**

**Alpha diversity indices of extreme RFI Charolais young bulls fed either a CS or GS diet.^1^**

| **Item** | **Species observed** | **Shannon** | **PD** |
| --- | --- | --- | --- |
| **Analysis of overall dataset** | | | |
| **Diet** | | | |
| CS | 624 | 5.9 | 35.2 |
| GS | 688 | 6.0 | 39.4 |
| *P-*value | 0.431 | 0.285 | 0.174 |
| **Year** | | | |
| 2019 | 776 | 6.1 | 44.5 |
| 2020 | 618 | 5.9 | 37.1 |
| *P-*value | 0.077 | 0.047 | 0.070 |
| **RFI** | | | |
| Low RFI | 685 | 6.0 | 39.2 |
| High RFI | 624 | 5.9 | 38.4 |
| *P-*value | 0.604 | 0.595 | 0.653 |
| **Analysis for each diet** | | | |
| **GS** | | | |
| **RFI** | | | |
| Low RFI | 701 | 6.0 | 39.4 |
| High RFI | 640 | 5.9 | 40.4 |
| *P-*value | 0.385 | 0.463 | 0.613 |
| **Year** | | | |
| 2019 | 837 | 6.2 | 45.8 |
| 2020 | 604 | 5.9 | 37.1 |
| *P-*value | 0.009 | 0.003 | 0.003 |
| **CS** | | | |
| **RFI** | | | |
| Low RFI | 672 | 6.0 | 38.5 |
| High RFI | 635 | 5.8 | 35.3 |
| *P-*value | 0.862 | 0.867 | 0.779 |
| **Year** | | | |
| 2019 | 640 | 5.9 | 35.9 |
| 2020 | 670 | 5.9 | 38.2 |
| *P-*value | 0.862 | 0.867 | 0.955 |

RFI = residual feed intake; GS = grass silage; CS = corn silage; PD = phylogenetic diversity.

^1^  Each value represents the median per experimental group (GS – Low RFI, *n* = 7; GS – High RFI, *n* = 8; CS – Low RFI, *n* = 8; CS – High RFI, *n* = 7). The median comparisons between the experimental groups were analyzed using the Wilcoxon rank sum test.

**Table S2.**

**Microbial functional category abundances (means of cpm ± SD) of Charolais young bulls fed either a GS or CS diet.^1^**

| **Parameters** | **GS** | **CS** | ***q*-values^2^** |
| --- | --- | --- | --- |
| **KEGG functional category** | | | |
| Carbohydrate metabolism | 297,000 ± 5,485 | 305,000 ± 5,464 | 0.704 |
| Energy metabolism | 234,000 ± 4,984 | 247,000 ± 3,417 | 0.250 |
| Amino acid metabolism | 170,000 ± 5,556 | 168,000 ± 6,884 | 0.839 |
| Nucleotide metabolism | 87,311 ± 1,874 | 78,642 ± 2,006 | 0.043 |
| Metabolism of cofactors and vitamins | 53,979 ± 2,963 | 55,576 ± 1,264 | 0.739 |
| Lipid metabolism | 39,141 ± 835 | 37,105 ± 1,201 | 0.651 |
| Metabolism of other amino acids | 32,685 ± 842 | 31,332 ± 695 | 0.704 |
| Glycan biosynthesis and metabolism | 23,234 ± 872 | 20,025 ± 676 | 0.043 |
| Xenobiotic biodegradation and metabolism | 23,008 ± 2,517 | 21,467 ± 539 | 0.739 |
| Biosynthesis of other secondary metabolites | 22,274 ± 761 | 19,593 ± 737 | 0.098 |
| Metabolism of terpenoids and polyketides | 16,996 ± 465 | 16,755 ± 619 | 0.788 |
| **COG functional categories** | | | |
| Carbohydrate transport and metabolism | 183,000±5,883 | 191,000±6,000 | 0.697 |
| Translation ribosomal structure and biogenesis | 181,000±3,976 | 178,000±5,264 | 0.877 |
| Amino acid transport and metabolism | 109,000±5,224 | 101,000±5,961 | 0.697 |
| Energy production and conversion | 83,268±1,158 | 92,493±2,568 | 0.049 |
| General function prediction only | 63,232±1,003 | 60,992±914 | 0.394 |
| Posttranslational modification protein turnover chaperones | 50,999±5,379 | 54,666±6,178 | 0.855 |
| Replication recombination and repair | 48,965±3,095 | 49,970±1,175 | 0.901 |
| Nucleotide transport and metabolism | 41,559±1,403 | 37,428±950 | 0.152 |
| Cell wall membrane envelope biogenesis | 36,840±716 | 34,800±671 | 0.152 |
| Inorganic ion transport and metabolism | 32,119±2,037 | 31,707±2,831 | 0.947 |
| Coenzyme transport and metabolism | 30,128±1,989 | 32,535±823 | 0.621 |
| Lipid transport and metabolism | 28,109±425 | 26,253±673 | 0.152 |
| Intracellular trafficking secretion and vesicular transport | 24,300±555 | 23,687±702 | 0.775 |
| Signal transduction mechanisms | 22,256±1,454 | 19,213±974 | 0.291 |
| Function unknown | 20,387±708 | 20,944±386 | 0.775 |
| Transcription | 19,133±997 | 18,221±382 | 0.704 |
| Cell cycle control cell division chromosome partitioning | 10,198±259 | 10,883±254 | 0.174 |
| Cell motility | 6,611±200 | 5,727±260 | 0.112 |
| Secondary metabolites biosynthesis transport and catabolism | 5,698±320 | 5,615±313 | 0.910 |
| Defense mechanisms | 3,524±81 | 4,159±107 | 0.003 |
| RNA processing and modification | 51±4 | 43±4 | 0.416 |
| Nuclear structure | 32±5 | 19±3 | 0.152 |

GS = grass silage; CS = corn silage; SD = standard deviation; cpm = counts per million reads.

^1^  Each value represents the mean of cpm per experimental group (GS – Low RFI, *n* = 8; GS – High RFI, *n* = 8; CS – Low RFI, *n* = 8; CS – High RFI, *n* = 8).

^2^ The *q*-value was obtained after false discovery rate (FDR) correction using the Benjamini-Hochberg procedure.

**Table S3.**

**Microbial functional category abundances (means of counts per million, cpm ± SD) of extreme RFI Charolais young bulls fed either a CS or GS diet.^1^**

| **KEGG functional category** | **High RFI** | **Low RFI** | ***P-*value** | ***q*-value** |
| --- | --- | --- | --- | --- |
| Carbohydrate metabolism | 306,000 ± 4,880 | 297,000 ± 5,966 | 0.262 | 0.704 |
| Energy metabolism | 241,000 ± 3,722 | 240,000 ± 5,264 | 0.842 | 0.842 |
| Amino acid metabolism | 165,000 ± 5,492 | 173,000 ± 6,808 | 0.405 | 0.704 |
| Nucleotide metabolism | 82,316 ± 2,138 | 83,658 ± 2,328 | 0.620 | 0.740 |
| Metabolism of cofactors and vitamins | 54,095 ± 1,594 | 55,460 ± 2,803 | 0.640 | 0.740 |
| Lipid metabolism | 38,494 ± 1,106 | 37,752 ± 1,019 | 0.618 | 0.740 |
| Metabolism of other amino acids | 32,325 ± 759 | 31,693 ± 815 | 0.575 | 0.740 |
| Biosynthesis of other secondary metabolites | 21,449 ± 899 | 20,419 ± 720 | 0.342 | 0.704 |
| Glycan biosynthesis and metabolism | 21,405 ± 833 | 21,854 ± 927 | 0.650 | 0.740 |
| Xenobiotic biodegradation and metabolism | 21,126 ± 479 | 23,349 ± 2,512 | 0.404 | 0.704 |
| Metabolism of terpenoids and polyketides | 17,081 ± 597 | 16,670 ± 488 | 0.572 | 0.740 |
| **COG functional category** | | | | |
| Carbohydrate transport and metabolism | 190,000 ± 5,597 | 184,000 ± 6,375 | 0.534 | 0.775 |
| Translation ribosomal structure and biogenesis | 182,000 ± 4,651 | 177,000 ± 4,585 | 0.405 | 0.755 |
| Amino acid transport and metabolism | 102,000 ± 4,934 | 108,000 ± 6,287 | 0.496 | 0.775 |
| Energy production and conversion | 90,320 ± 2,761 | 85,441 ± 1,533 | 0.084 | 0.291 |
| General function prediction only | 62,266 ± 1,171 | 61,958 ± 796 | 0.828 | 0.910 |
| Posttranslational modification protein turnover chaperones | 51,034 ± 5,643 | 54,631 ± 5,939 | 0.672 | 0.855 |
| Replication recombination and repair | 48,525 ± 1,282 | 50,410 ± 3,038 | 0.550 | 0.775 |
| Nucleotide transport and metabolism | 38,459 ± 1,132 | 40,529 ± 1,420 | 0.236 | 0.562 |
| Cell wall membrane envelope biogenesis | 35,370 ± 753 | 36,271 ± 712 | 0.325 | 0.697 |
| Inorganic ion transport and metabolism | 31,374 ± 2,299 | 32,452 ± 2,616 | 0.757 | 0.901 |
| Coenzyme transport and metabolism | 30,704 ± 924 | 31,959 ± 1,980 | 0.562 | 0.775 |
| Lipid transport and metabolism | 27,430 ± 706 | 26,933 ± 491 | 0.544 | 0.775 |
| Intracellular trafficking secretion and vesicular transport | 24,056 ± 617 | 23,931 ± 658 | 0.885 | 0.940 |
| Signal transduction mechanisms | 20,964 ± 1,518 | 20,508 ± 1,029 | 0.789 | 0.910 |
| Function unknown | 20,586±460 | 20,746 ± 669 | 0.831 | 0.910 |
| Transcription | 18,355 ± 514 | 18,999 ± 943 | 0.515 | 0.775 |
| Cell cycle control cell division chromosome partitioning | 10,612 ± 280 | 10,466 ± 261 | 0.662 | 0.855 |
| Cell motility | 6,129 ± 275 | 6,209 ± 240 | 0.813 | 0.910 |
| Secondary metabolites biosynthesis transport and catabolism | 5,401 ± 158 | 5,912 ± 409 | 0.234 | 0.562 |
| Defense mechanisms | 3,890 ± 137 | 3,792 ± 111 | 0.467 | 0.775 |
| RNA processing and modification | 49 ± 4 | 45 ± 4 | 0.520 | 0.775 |
| Nuclear structure | 26 ± 5 | 26 ± 4 | 0.967 | 0.967 |

RFI = residual feed intake; GS = grass silage; CS = corn silage; SD = standard deviation; cpm = counts per million reads.

^1^  Each value represents the mean of cpm per experimental group (GS – Low RFI, *n* = 8; GS – High RFI, *n* = 8; CS – Low RFI, *n* = 8; CS – High RFI, *n* = 8).

^2^ The q-value was obtained after false discovery rate (FDR) correction using the Benjamini-Hochberg procedure.

**Table S4.**

**Summary of information of previous ten studies, limited to beef cattle, mostly rumen; meta-taxonomic data, illumina sequencing and animals with extreme RFI phenotype.**

| **N°** | **Metadata** | |  |  |  | |  | **Results** |  | **References** |
| --- | --- | --- | --- | --- | --- | --- | --- | --- | --- | --- |
|  | **Age category** | **Breed** | **Sex** | **Diets^1^** | **Sample type** | **Sampling time** | **DA methods** | **Beta-diversity** | **DA of taxa** |  |
| 1 | Steers | Nellore | M | High-concentrate | Rumen | Pre-feeding | LDA LEfSe | Not differed | LRFI: ↗*Lachnospiraceae*^2^, *Ruminococcaceae* and *Christensenellaceae* | Lopes et al. (2021) |
| 2 | Steers | Charolais | M | High concentrate | Rumen | Post-feeding (2 and 4 h) | MaAsLin2 | Not differed | LRFI: ↗*Mogibacteriaceae*, *Methanomassiliicoccaceae*, *Ruminobacter* and *Lactobacillales* | McGovern et al. (2020) |
| 3 | Steers | Nellore | M | High concentrate | Rumen | Pre-feeding | Ven diagram | Not differed | LRFI; ↗*Lachnospiraceae*^2^, *Ruminococaceae*, and *Bacteroidales* | Lopes et al. (2019) |
| 4 | Steers | Crossbred | M | High concentrate | Rumen | Pre-feeding | LDA LEfSe | Not differed | HRFI: ↗*Lachnospiraceae*^2^, *Lactobacillaceae*, and *Veillonellaceae* | Li and Guan (2017) |
| 5 | Steers | Angus | M | High concentrate | Feces | Pre-feeding | ANOVA | Not differed | LRFI: ↗*Ruminococcaceae* | Welch et al. (2020) |
| 6 | Bull | Simmental | M | High concentrate | Rumen | Pre-feeding | Wilcoxon rank sum test | Not differed | LRFI: ↗*Fibrobacteres*, *Cyanobacteria* and *Tenericutes* | McGovern et al. (2018) |
| 7 | Steers | Angus | M | Low concentrate | Rumen | Not reported | Random Forest classification | Not differed | LRFI: ↗*Flavobacteriia* and *Fusobacteriia* | Clemmons et al. (2019) |
| 8 | Bull | Nellore | M | Low concentrate | Rumen | Pre-feeding | ANCOM and MaAsLin2 | Not differed | LRFI: ↗*Rikenellaceae RC9* | Andrade et al. (2022) |
| 9 | Heifers | Angus | F | Medium concentrate | Rumen | Pre-feeding | T-Test | Differed | LRFI: ↗*Proteobacteria*, *Rikenellaceae*, *Ruminococcaceae*, and *Lachnospiraceae*^2^ | Liu et al. (2022) |
| 10 | Heifers | Angus | F | Medium concentrate | Rumen | Not reported | LDA LEfSe | Differed | LRFI: ↗*Lachnospiraceae*^2^, *RF32*, *Neisseriaceae*, and *Shutlewortia* | Clemmons et al. (2022) |

RFI = residual feed intake; DA = differential abundance; F = female; M = male.

^1^ High concentrate diet, means more than 60% of concentrate in total mixed ration; Medium concentrate diet, means 50% concentrate + 50% forage; Low concentrate diet, means more 60% of forage in total mixed ration.

^2^ Most abundant taxa in Low RFI, which have similarities to our results.

**References**

Andrade BGN, Bressani FA, Cuadrat RRC, Cardoso TF, Malheiros JM, de Oliveira PSN, et al. Stool and Ruminal Microbiome Components Associated With Methane Emission and Feed Efficiency in Nelore Beef Cattle. Front Genet 2022;13. https://doi.org/10.3389/fgene.2022.812828.

Bes A, Nozière P, Renand G, Rochette Y, Guarnido-Lopez P, Cantalapiedra-Hijar G, et al. Individual methane emissions (and other gas flows) are repeatable and their relationships with feed efficiency are similar across two contrasting diets in growing bulls. Animal 2022;16:100583. https://doi.org/10.1016/j.animal.2022.100583.

Blackburn TH, Hungate RE. Succinic Acid Turnover and Propionate Production in the Bovine Rumen. Appl Microbiol 1963;11:132–5. https://doi.org/10.1128/am.11.2.132-135.1963.

Clemmons BA, Martino C, Powers JB, Campagna SR, Voy BH, Donohoe DR, et al. Rumen Bacteria and Serum Metabolites Predictive of Feed Efficiency Phenotypes in Beef Cattle. Sci Rep 2019;9:19265. https://doi.org/10.1038/s41598-019-55978-y.

Clemmons BA, Mulon *P-*Y, Anderson DE, Ault-Seay TB, Henniger MT, Schneider LG, et al. Ruminal Bacterial Communities and Metabolome Variation in Beef Heifers Divergent in Feed Efficiency. Ruminants 2022;2:282–96. https://doi.org/10.3390/ruminants2020019.

Dehority BA. Pectin-fermenting Bacteria Isolated from the Bovine Rumen. J Bacteriol 1969;99:189–96. https://doi.org/10.1128/jb.99.1.189-196.1969.

Li F, Guan LL. Metatranscriptomic Profiling Reveals Linkages between the Active Rumen Microbiome and Feed Efficiency in Beef Cattle. Appl Environ Microbiol 2017;83. https://doi.org/10.1128/AEM.00061-17.

Liu Y, Wu H, Chen W, Liu C, Meng Q, Zhou Z. Rumen Microbiome and Metabolome of High and Low Residual Feed Intake Angus Heifers. Front Vet Sci 2022;9. https://doi.org/10.3389/fvets.2022.812861.

Lopes DRG, La Reau AJ, Duarte M de S, Detmann E, Bento CBP, Mercadante MEZ, et al. The Bacterial and Fungal Microbiota of Nelore Steers Is Dynamic Across the Gastrointestinal Tract and Its Fecal-Associated Microbiota Is Correlated to Feed Efficiency. Front Microbiol 2019;10. https://doi.org/10.3389/fmicb.2019.01263.

Lopes DRG, de Souza Duarte M, La Reau AJ, Chaves IZ, de Oliveira Mendes TA, Detmann E, et al. Assessing the relationship between the rumen microbiota and feed efficiency in Nellore steers. J Anim Sci Biotechnol 2021;12:79. https://doi.org/10.1186/s40104-021-00599-7.

Mahmood M, Khiaosa-ard R, Zebeli Q, Petri RM. Betaine Modulates Rumen Archaeal Community and Functioning during Heat and Osmotic Stress Conditions In Vitro. Archaea 2020;2020:1–17. https://doi.org/10.1155/2020/8875773.

McGovern E, Kenny DA, McCabe MS, Fitzsimons C, McGee M, Kelly AK, et al. 16S rRNA Sequencing Reveals Relationship Between Potent Cellulolytic Genera and Feed Efficiency in the Rumen of Bulls. Front Microbiol 2018;9. https://doi.org/10.3389/fmicb.2018.01842.

McGovern E, McGee M, Byrne CJ, Kenny DA, Kelly AK, Waters SM. Investigation into the effect of divergent feed efficiency phenotype on the bovine rumen microbiota across diet and breed. Sci Rep 2020;10:15317. https://doi.org/10.1038/s41598-020-71458-0.

Oosterveld A, Beldman G, Schols HA, Voragen AGJ. Arabinose and ferulic acid rich pectic polysaccharides extracted from sugar beet pulp. Carbohydr Res 1996;288:143–53. https://doi.org/10.1016/S0008-6215(96)90791-0.

Patterson JA, Hespell RB. Trimethylamine and methylamine as growth substrates for rumen bacteria andMethanosarcina barkeri. Curr Microbiol 1979;3:79–83. https://doi.org/10.1007/BF02602436.

Sun X, Cheng L, Jonker A, Munidasa S, Pacheco D. A Review: Plant Carbohydrate Types—The Potential Impact on Ruminant Methane Emissions. Front Vet Sci 2022;9. https://doi.org/10.3389/fvets.2022.880115.

Thoetkiattikul H, Mhuantong W, Laothanachareon T, Tangphatsornruang S, Pattarajinda V, Eurwilaichitr L, et al. Comparative Analysis of Microbial Profiles in Cow Rumen Fed with Different Dietary Fiber by Tagged 16S rRNA Gene Pyrosequencing. Curr Microbiol 2013;67:130–7. https://doi.org/10.1007/s00284-013-0336-3.

Wang Lijun, Li Y, Zhang Y, Wang Lihua. The Effects of Different Concentrate-to-Forage Ratio Diets on Rumen Bacterial Microbiota and the Structures of Holstein Cows during the Feeding Cycle. Animals 2020;10:957. https://doi.org/10.3390/ani10060957.

Welch CB, Lourenco JM, Davis DB, Krause TR, Carmichael MN, Rothrock MJ, et al. The impact of feed efficiency selection on the ruminal, cecal, and fecal microbiomes of Angus steers from a commercial feedlot. J Anim Sci 2020;98. https://doi.org/10.1093/jas/skaa230.
